# Fine needle aspirates of kidneys: a promising tool for RNA sequencing in native and transplanted kidneys

**DOI:** 10.1186/s12882-018-1012-4

**Published:** 2018-09-05

**Authors:** Øystein Eikrem, Tedd C. Walther, Arnar Flatberg, Vidar Beisvag, Philipp Strauss, Magnus Farstad, Christian Beisland, Even Koch, Thomas F. Mueller, Hans-Peter Marti

**Affiliations:** 1 Department of Clinical Medicine, Nephrology, University of Bergen, Bergen, Norway; 2 Department of Medicine, Haukeland University Hospital, Bergen, Norway; 3 Department of Clinical and Molecular Medicine, Norwegian University of Science and Technology, Trondheim, Norway; 4 Department of Urology, Haukeland University Hospital, Bergen, Norway; 5 Division of Nephrology, Department of Medicine, University Hospital of Zurich, Zurich, Switzerland

**Keywords:** Core biopsy, Fine needle aspiration, Gene expression, Rejection, RNA sequencing

## Abstract

**Background:**

Transcriptome analysis is emerging as emerging as a promising tool to enhance precision of diagnosis and monitoring in solid organ transplantation. Clinical progress has however been hampered by the current reliance on samples from core needle biopsies. This proof-of-principle study examined whether fine needle aspirates, being less invasive, permit the ascertainment of the identical molecular information as core biopsies.

**Methods:**

We collected fine needles aspirates from various needle sizes (G19, 21, 23, 25) and the corresponding core biopsies (G16 needle) of non-tumor tissue of full nephrectomy specimens from patients suffering from clear cell renal cell carcinoma (*n* = 11). RNA expression patterns of two gene sets (156 genes) were executed using targeted RNA sequencing in samples from fine needle vs. core needle samples. A subgroup of kidneys (*n* = 6) also underwent whole transcriptome RNA sequencing from core biopsies of tumor and peri-tumoral normal tissue (Tru Seq RNA Access, Illumina).

**Results:**

Samples from all needle sizes except two G25 aspirates yielded RNA potentially suitable for sequencing of both gene sets. The mRNA expression patterns of the two gene sets were highly correlated between fine needle aspirates (G23) and corresponding (G16) core biopsies (*r* = 0.985 and 0.982, respectively). This close correlation was further documented by heat map, Principal Component Analyses (PCA) and whole transcription RNA sequencing. The similarity between fine neddle aspirates and core needle biopsies was additionally confirmed in the subgroup with complete RNA sequencing.

**Conclusions:**

Fine needle biopsies yield similar genomic information to core needle biopsies. The less invasive nature of fine needle biopsies may therefore permit more frequent molecular monitoring and a more targeted use of core needle biopsies in native and especially in transplanted kidneys.

## Background

The diagnosis of rejection in renal transplantation is currently reliant on core needle biopsies given the lack of specificity of renal function measurements in blood or urine. Core biopsies are generally accepted to be safe but are expensive, labor-intensive, and can be accompanied by serious complications that may require hospitalization, such as gross hematuria requiring blood transfusions as well as even surgical interventions or angiographic embolizations (1–2.5% of kidney biopsies) [1, 2]. Frequent monitoring of transplanted kidney status using repeated core needle biopsies is therefore not routinely feasible.

A less-invasive method of monitoring allograft status would facilitate understanding and management of allograft dysfunction. Such a tool would be especially relevant where protocol biopsies are recommended to detect subclinical T cell or antibody-mediated rejection, prior to overt functional allograft deterioration, or where response to therapy may have been suboptimal in routine practice [3].

Molecular analyses are emerging as important techniques to complement or in certain cases to even replace tissue histology in diagnosis of transplant dysfunction. Robust transcript sets have been identified which predict future tissue alterations and/or reflect early acute or chronic allograft rejection in renal allografts [4, 5]. A set of 13 transcripts were found to be predictive of fibrosis at 1 year and subsequent loss of allograft function [6]. Endothelial cell-derived transcript expression was shown to reflect active antibody-mediated microvascular injury and poor transplant outcome despite absence of histologic C4d staining [7]. A gene set has been described which differentiated polyomavirus-associated nephropathy from acute renal allograft rejection [8]. In each of these circumstances, the molecular diagnosis provided relevant clinical information not revealed by histology alone. Gene profiling has also been extended to other organs and disease processes owing to the highly stereotyped and organ-unspecific molecular patterns associated with inflammation and immune responses [9].

Given the inherent risk of repeated core needle biopsies, but the high potential clinical value of obtaining transcriptome information, we have investigated whether FNA, being less invasive, yields equivalent molecular information to regular core needle biopsies. In this proof-of-principle study we compared RNA sequencing results from two well defined gene sets (cell cycle and Wnt panels) in aspirates and biopsies from nephrectomy specimens.

## Methods

### Patients and tissue samples

Kidney tissue samples were obtained from patients (*n* = 11) with suspected renal cell carcinoma at the time of radical nephrectomy. All patients had normal kidney function (eGFR > 60 ml/min/1.73m^2^) and had undergone total nephrectomy. Key characteristics of the patients are outlined in Table 1. One 16 gauge (G16) core biopsy and four fine needle aspirate samples (FNA) using 19-, 21-, 23- and 25-gauge needles were obtained with one pass (attempt) per individual biopsy or FNA from normal appearing kidney tissue as far as possible away from the tumor. Kidneys with warm ischemia time over 2 hours were excluded. Ethics approval was granted by the regional ethics committee of the Western Norway Regional Health Authority (REK vest); approval number: 78–05. Written informed consent was obtained from all patients.

**Table 1:** Patient/sample overview at the time of nephrectomy (*n* = 11)

Patient	Targeted-panel seq Core biopsy (G16) [healthy tissue]	Targeted-panel seq FNA (G23) [healthy tissue]	Whole transcriptome RNAseq (G16), Tumor [tumor biopsy]	Whole transcriptome RNAseq (G16), Non tumor [healthy tissue]
39 N	X	X	X	X
42 N	X	X		
44 N	X	X	X	X
47 N	X	X		
49 N	X	X		
50 N	X	X	X	X
57 N	X	X	X	X
64 N	X	X	X	X
65 N	X	X	X	X
66 N	X	X		
69 N	X	X		

Seven males and four females with a mean age of 64 years (95% CI: 58–70) were included. A subset of patients (*n* = 6) underwent both two-panel mRNA sequencing and full mRNA sequencing

Concurrently harvested kidney biopsies (G16) from the tumor and adjacent normal tissue from a nested subset (*n* = 6) of the total group of subjects (*n* = 11), stored as formalin-fixed, paraffin-embedded (FFPE) tissues, had previously been analyzed by whole transcriptome RNA sequencing (TruSeq RNA Access kit®, Illumina, USA) (Table 1) [10, 11]. The complete transcriptome RNA sequencing data from these patients (*n* = 6) were included to investigate potential differences between whole transcriptome and targeted sequencing.

### RNA extraction and analysis

Core biopsy samples (G16) were immediately placed in RNA later® (Qiagen, Netherlands). FNA samples (G19–25) were placed directly into 700 μl of Qiazol lysis buffer (Qiagen, Netherlands). Samples were stored at − 80 °C.

Core needle kidney biopsy samples were weighed and cut into pieces of approximately 5 mg. Homogenization of biopsy cores and FNA samples was performed in 700 μl Qiazol lysis buffer with ceramic beads. All samples were homogenized for 3 × 10 seconds at 6800 rpm on a Precellys homogenizer (Bertin Technologies, USA). Total RNA was extracted using the miRNeasy Micro kit (Qiagen, Netherlands). RNA was stored at -80C until further use for RNA sequencing.

RNA concentration was measured using a NanoDrop 1000 spectrophotometer (Thermo Fisher Scientific, USA). RNA integrity was assessed using the Agilent RNA 6000 Nano kit on a 2100 Bioanalyzer (Agilent Technologies, USA). The DV200 value (the percentage of RNA fragments longer than 200 nucleotides) was used to reflect RNA quality for subsequent sequencing from FFPE tissues [10, 11]. A minimum DV200 value of 30% is recommended and required for RNA sequencing [12]. RNA integrity number (RIN) values were also determined.

### RNA sequencing

RNA sequencing and analyses were conducted using targeted RNA expression, heatmaps and PCA. RNA sequencing was performed using two different predesigned TruSeq Targeted RNA panels from Illumina©. Panel sequencing was selected because, if successful, sequencing of defined gene panels (e.g. related to rejection processes) is much more likely to be integrated into routine clinical practice than full transcriptome sequencing.

To test the potential utility of FNA-derived RNA sequencing, gene sets with relevance in kidney allografts were selected. The TruSeq Targeted RNA Expression “Cell Cycle Panel” includes 63 genes/transcripts participating in the cell cycle and DNA replication. The complete gene set of this expression panel is available at http://www.support.illumina.com/content/illumina-marketing/en/products/truseq-targeted-rna-expressioncell-cycle-panel.html. This panel was selected because cell cycle genes would reflect injury-repair mechanisms in the renal allograft [13]. The TruSeq Targeted RNA Expression “Wnt Panel” includes assays for 93 genes/transcripts involved in upstream and downstream signal transduction in the Wnt signalling pathway, including transcription factors as well as target genes. The complete gene list of this expression panel is available at http://www.support.illumina.com/content/illumina-marketing/en/products/truseq-targeted-rna-expressionwnt-panel.html. This panel was selected given its general importance in biological processes, and because the wnt pathway has been implicated in chronic renal allograft injury [14].

Sequencing libraries were generated according to manufacturer’s instructions (Illumina, Inc., San Diego, USA) with RNA normalized to inputs of 200 ng total RNA from each sample. Equal amounts of libraries were pooled (normalized) to a final concentration of 18 pM and subjected to cluster and single end read sequencing.

Sequencing was performed for 50 cycles using MiSeq Reagent Kit v3 on a MiSeq® instrument (Illumina, Inc., San Diego, USA). Base calling was done on the instrument and Fastq files were generated using bcl2fastq v.2.18 with default settings for the RNA-seq data. Transcript expression values were determined using the Salmon software [15] with the GRCh37 transcriptome downloaded from the Ensembl database (http://www.ensembl.org/).

Gene counts from the 2 gene sets were combined in the tximport package (https://bioconductor.org/packages/release/bioc/html/tximport.html). Genes with less than two counts total were filtered out prior to analysis.

Variance transformed expression data was generated with the DESeq2 package (https://bioconductor.org/packages/release/bioc/html/DESeq2.html) using the rlog transform on count data. Heatmaps for expression data were generated using the pheatmap package (https://cran.r-project.org/web/packages/pheatmap) and hierarchical clustering of genes and samples in the heatmap was generated using the Ward’s clustering method and correlation distances. Principal Component Analysis (PCA) was performed on variance transformed data (rlog) and visualized using the ggfortify package (https://cran.r-project.org/web/packages/ggfortify). The average expression values for the G16 biopsy (*n* = 11) and G23 FNA (*n* = 11) gene panel data, as well as for the prior G16 biopsy (*n* = 6) whole transcriptome data, were entered into a generalized pair plot using the GGally package (https://cran.r-project.org/web/packages/GGally).

The datasets created during and/or analysed during the current study are available from the corresponding author upon reasonable request.

## Results

### RNA quantity and quality obtained from fine needle aspirates (FNA) and core needle biopsies is sufficient and high

A subset of nephrectomy samples (*n* = 6) underwent both, prior sequencing of whole transcriptome RNA (as recently published [11]) and sequencing of two targeted RNA expression panels from core biopsies and FNAs. The results from the whole transcriptome RNA sequencing (*n* = 6) served as an internal control and comparator (*n* = 6) to detect differences between whole transcriptome and targeted panel sequencing.

**Fig. 1:**
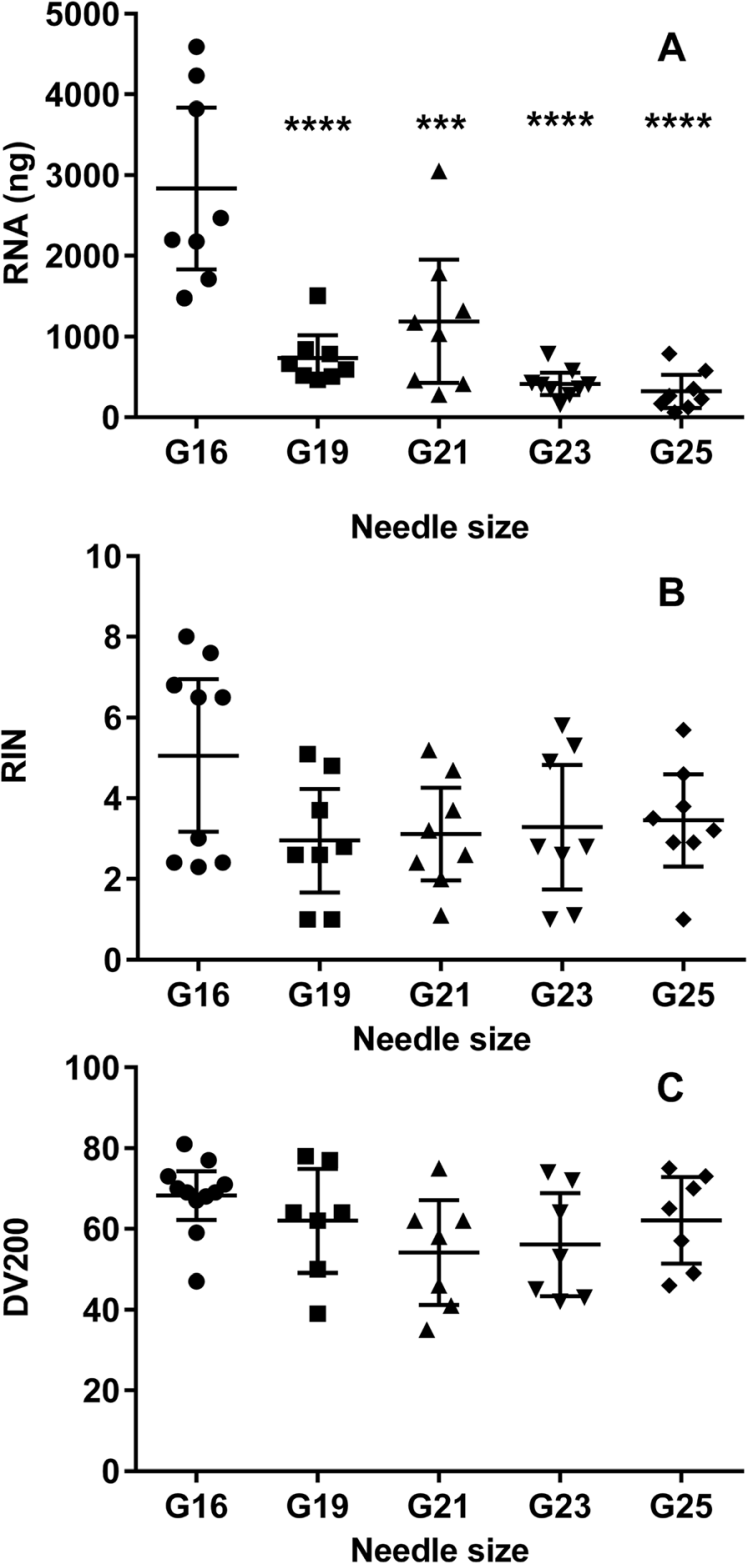
RNA yield and RNA quality of FNA samples (G19-G25) and of corresponding core biopsy samples (G16). **a**. RNA yield (ng/sample) was higher in G16 biopsies compared to all FNA (G19–25) samples (***p = 0,0003; ****p < 0,0001). **b**. RNA quality by RIN values, and **c**. RNA quality by DV200 values

RNA quality for sequencing was expressed by the RIN and the DV200 values. The quantity (Fig. 1a) and quality (Fig. 1b and c) of extracted RNA from the 11 samples stratified by different needle sizes is shown in Fig. 1.

The mean DV200 values (95% CI) were 56% (43–69) for G23 FNA samples and 68% (62–74) for G16 core biopsy samples. The DV200 percentages well exceed the 30% minimum required for RNA sequencing [12]. The RIN-values were 3.3 (1.7–4.8) and 5.1 (3.2–6.9) for the FNA samples and G16 biopsies, respectively. Importantly, RIN-values and DV200 numbers were not significantly different between any of the needle sizes (G16-G25), as assessed by one-way ANOVA with Tukey’s multiple comparisons test (Fig. 1); adj. *p* ≥ 0.13 for all comparisons.

These RIN values reflected the expected RNA degradation from our FFPE tissues. However, RIN values derived from FFPE samples are not a reliable predictor of RNA quality or of successful library preparation using the Illumina TruSeq RNA Access Kit®, as investigated by the vendor, https://www.illumina.com/documents/products/technotes/technote-trusec-rna-access.pdf and recently described [10]. Mean RNA fragment sizes reflected by the DV200 percentages and not RIN values were therefore used to asses RNA quality from FFPE tissues for the subsequent RNA sequencing library preparation [10, 12, 16, 17].

As expected, one-way ANOVA with Tukey’s multiple comparisons test revealed a significantly lower RNA yield from all FNA compared to G16 core biopsy samples (Fig. 1). However, there were no significant differences in the RNA yield between any of the fine needle aspirates (G19-G25); adj. *p* ≥ 0.11 for all comparisons.

FNA from needles of all tested sizes consistently yielded sufficient quantities of RNA for sequencing with the exception of two G25 aspirates, which would have permitted sequencing of only one gene panel. Also, the total RNA yield from some of these G25 size aspirations was, however, at the threshold of 50 ng/sample required for RNA input for both sequencing panels according to Illumina (http://www.support.illumina.com/content/illumina-marketing/en/products/truseq-targeted-rna-expressionwnt-panel.html and http://www.support.illumina.com/content/illumina-marketing/en/products/truseq-targeted-rna-expression-cell-cycle-panel.html). RNA sequencing analyses were therefore performed using the G23 FNA aspirates, as the yield was reliable and this needle size is routine used for FNA in clinical practice.

### High degree of similarity in gene expression as depicted in a heatmap

**Fig. 2:**
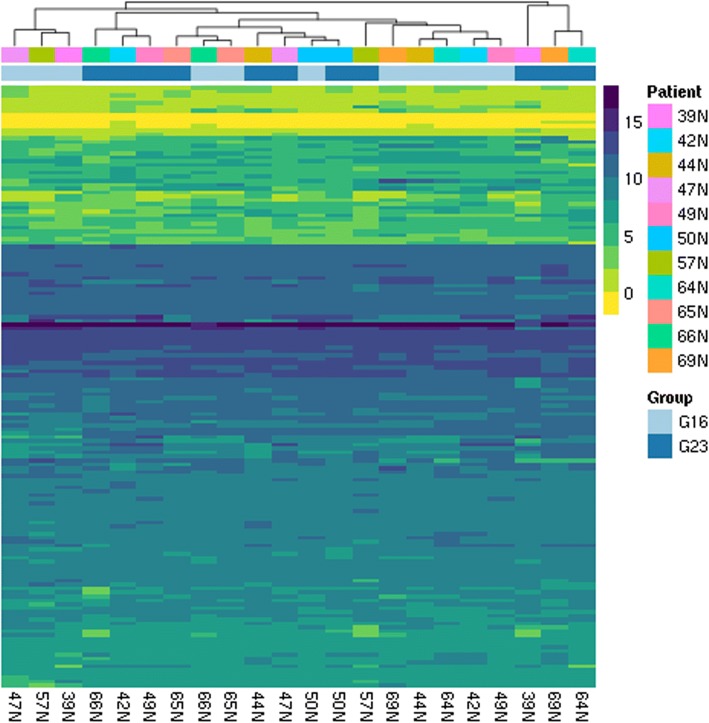
Heatmap. Hierarchical clustering of genes and samples generated using the Ward’s clustering method and correlation distances between samples and Euclidean distances between genes. The heatmap cells are colored proportional to rlog expression values

The heatmap (Fig. 2) depicts the transcript levels of the total of 156 genes included in both panels (cell cycle and Wnt pathway)**.** Overall the gene expression pattern obtained by RNA sequencing shows a high degree of similarity between the 11 FNA (G23) and the corresponding 11 core needle biopsy samples (G16); samples of some patients (e.g. 50 N) clustered together closely, others did not.

### Principal component analyses detecting similarity and dissimilarity among the datasets

**Fig. 3:**
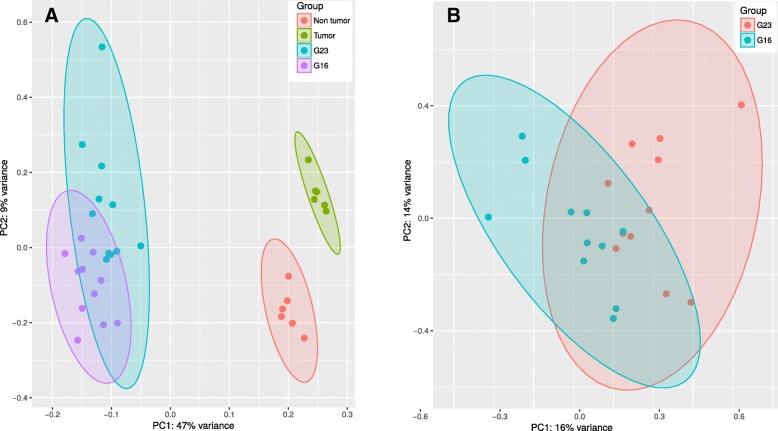
Principal Component Analyses (PCA). The first two principal components from a Principal Component Analysis using rlog transformed expression values. **a**. Principal Component Analysis (PCA) of samples from all four groups described in Table 1. The first principal component (PC1, x-axis) explains 47% of the variation in the data while the second principal component (PC2, y-axis) increases total explained variation to 56%. Confidence ellipsis at 99% is drawn for each group. **b**. Principal component analysis (PCA) of G23 vs. G16. The first principal component (PC1, x-axis) explains 16% of the variation in the data while the second principal component (PC2, y-axis) increases total explained variation to 30%. Confidence ellipsis at 99% is drawn for each group

The Principal Component Analysis (PCA) describes the variance across the four groups (Table 1). Figure 3a depicts a clear separation between i) the two sample sets analyzed with targeted RNA sequencing (G23, *n* = 11; and G16, *n* = 11), and ii) the two sample sets from the subset of patients with additional complete RNA sequencing of renal cell carcinoma tissues (“Tumor”, *n* = 6) and their adjacent renal tissue (“Non tumor”, *n* = 6). The two components (PC1 and PC2) of the PCA indicate that the expression patterns of the whole-genome based tumor tissues differs from non-tumor tissues, but taken together these two whole genome groups are much more dissimilar to the targeted sequencing samples. Therefore, the internal comparator group with complete RNA sequencing underscores the similarity between fine (G23) and core needle biopsies (G16) with targeted sequencing. The FNA (G23) and core biopsy (G16) groups show a high degree of overlap, indicating similar expression patterns in the sequencing results of the two gene panels.

The validity of RNA sequencing obtained from FNA samples is further underlined by the PCA in Fig. 3b, which emphasizes the lack of dissimilarity in RNA expression between the G23 and G16 samples, with low variance across the two dimensions PC1 and PC2. RNA expression in FNA and core needle biopsies therefore overlap closely.

### Significant correlation in gene expression between fine needle and core needle biopsies

To test the true utility of FNA against current standard core biopsy samples, we analyzed the correlation of gene expression between the two gene panels measured by sequencing in each of the three different sample groups: 11 FNA samples (G23), 11 core needle biopsies (G16) and 6 non-tumor whole genome tissue samples.

**Fig. 4:**
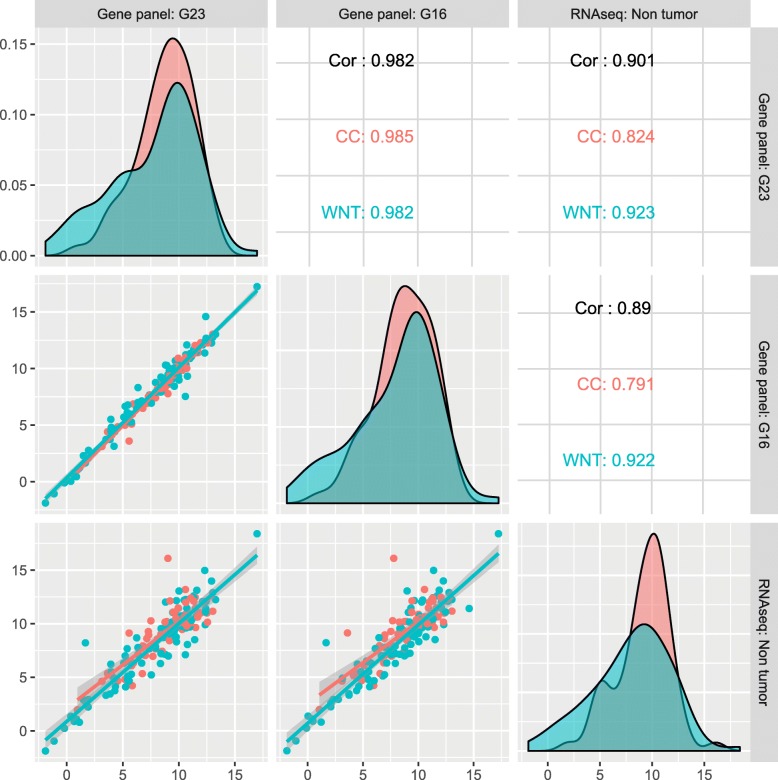
Correlations of gene expression. The x- and y-axis in each scatterplot represent average rlog transformed expression values for G16, G23 and non-tumor samples. The individual points are colored by the gene panel origin. The diagonal plots are density curves for the individual points and Pearson correlations are given for all genes (Cor; black), the WNT genes (WNT; green) and the Cell cycle genes (CC; magenta)

Figure 4 shows the high correlation for the mRNA expression patterns of the “cell cycle” (*n* = 63) and “Wnt” panel (*n* = 93) genes between the FNA and the respective core biopsies (*r* = 0.982 for all genes, 0.985 for cell cycle, and 0.982 for WNT panel, resp.). A minority of genes (*n* = 28) from the total of 156 genes were differentially expressed between the G23 and G16 groups, including genes such as MMP-9, MMP-2, IL-6, and FOXN1 which play a role in inflammatory or immunologic processes (data not shown)**;** this variability could be explained by the respective G23 and G16 needle location. The correlation of mRNA expression patterns between G23 and G16 samples and the respective results of the fully sequenced group (“Non tumor”, *n* = 6) was lower (r values between 0.791 and 0.923).

## Discussion

This study demonstrates that RNA sequencing of FNA samples provides equivalent molecular information to that of core needle biopsy samples. These proof of principle findings may justify a less invasive approach to immune monitoring in organ transplantation, which thus far has heavily relied on full core biopsies.

Currently percutaneous FNA is often performed for cancer diagnosis in various solid organs, such as breast, liver, and thyroid gland as well as in accessible lymph nodes [18]. FNAs are much easier to perform than core needle biopsies and are associated with significantly fewer complications. A recent metaanalysis of more than 4500 patients undergoing core-needle biopsy and/or FNA for thyroid cancer diagnosis reported an essentially 0 % complication rate [18]. In addition, cessation of oral anticoagulation or anti-platelet agents might not be necessary before fine needle aspiration [19, 20]. Importantly also, patients with potentially malignant renal masses, who have many comorbidities (e.g. cardiac events necessitating stents and platelet inhibitors) and a high operative risk, could benefit from the less invasive initial fine needle approach that includes transcriptome analyses. Indeed, a recent study demonstrated very good diagnostic accuracy - and a synergistic diagnostic advantage - of both FNA and core needle biopsy for renal masses in native kidneys [21]. Similar evidence in renal transplantation is lacking.

The present study demonstrates that sufficient quantities of RNA suitable for RNA sequencing can be obtained with a wide variety of fine needle sizes. Moreover, the comparative analyses indicate excellent correlation of mRNA expression patterns abundances obtained by FNA and full core biopsies. The internal comparator group with complete RNA sequencing further highlighted the similarity between fine and core needle biopsies with targeted sequencing.

This study has several important strengths. Current FNA and core biopsy samples were obtained under direct vision after nephrectomy and permitting stringent comparability of RNA expression from the various procedures taken from the same tissue area. The quality and quantity of RNA obtained by FNA was robust and therefore strongly suggest that FNA may be a viable diagnostic tool in selected patients. Despite these encouraging findings however, our study has several limitations that must be acknowledged. Samples were obtained from explanted kidneys which were no longer perfused, therefore potential confounding of FNA reproducibility by circulating blood cells as compared with core biopsy samples cannot be addressed. Comparative studies in perfused organs are required to assess the influence of peripheral blood and biopsy/aspiration site on the results. Clinical complications could not be assessed in this study, however a proof-of-principal study such as ours is a necessary ethical pre-requisite prior to consideration of clinical studies. Further limitations to our study include the facts that we provide panel sequencing and not complete RNA sequencing of the whole genome and that we did not determine the variability of RNA expression between multiple FNA aspirates and the same biopsy. Moreover, FNA is not able to deliver the transcriptome of individual cell types and a detailed pathology report cannot be assigned to an FNA. In addition, as with all transcriptome studies, it remains to be shown if mRNA results of our selected panel genes can be extrapolated to the respective gene product on the protein level and to other genes and pathways.

Transcriptome analysis has not yet reached the clinical mainstream and therefore the true clinical impact of facilitated transcriptome/RNA monitoring also requires prospective studies. It could be argued that the current hesitation in performing multiple biopsies in the same patient may have hindered progress in understanding the clinical value of the transcriptome. The use of FNA may therefore accelerate understanding of its clinical relevance. The close correlation of gene expression from RNA obtained with FNA as compared with core biopsies, as well as the relative safety, justifies further investigation and confirmation of the value of FNA in prospective clinical studies.

It is important to acknowledge however that FNA is not an adequate source for histological investigations. Therefore, we do not suggest that FNA can replace all histologic analysis, which remains important for diagnosis of disease recurrence, borderline rejection changes, viral infections etc. We continue to support the need for histology especially for the first biopsy to ensure that unexpected and transplant-unrelated diagnoses are not missed. It is conceivable however that FNA could replace protocol biopsies, especially where allograft function appears normal. More frequent monitoring with serial protocol FNA in immunologically high-risk kidney allografts might detect rejection processes earlier than histological changes and/or an overt decline in kidney function. Candidate gene panels for transplant rejection have already been defined [22, 23]. FNA would also be an attractive procedure for follow up monitoring of tissue response to therapy after a core biopsy baseline histologic and transcriptome diagnosis is made. Availability of diagnosis using FNA could optimize timing and reduce frequency of indication of core needle biopsies not only in kidney but also in other transplanted organs.

## Conclusions

In conclusion, fine needles aspirates reproducibly reflect molecular information in the kidney. FNA may therefore provide a simple and safe tool to monitor native and especially transplanted kidneys by supplementing the more invasive core needle biopsies and could provide accessible information to guide clinical decision making.

## Abbreviations

ccRCC: Clear cell renal cell carcinoma
eGFR: Estimated glomerular filtration rate
FNA: Fine needle aspirates
PCA: Principal component analysis
